# Achieving a Large Net “Negative Electron Affinity” on Diamond (100) via Molecular Oxygen and Lithium Functionalization

**DOI:** 10.1021/acsami.5c20029

**Published:** 2026-01-27

**Authors:** Ramiz Zulkharnay, William Greenwood, Adam Wood, Jude Laverock, Neil A. Fox

**Affiliations:** † School of Chemistry, 1980 University of Bristol, Cantock’s Close, Bristol BS8 1TS, U.K.; ‡ School of Physics, H.H. Wills Physics Laboratory, University of Bristol, Tyndall Avenue, Bristol BS8 1TL, U.K.

**Keywords:** diamond, oxidation, lithiation, negative electron affinity, surface modification, thermal activation

## Abstract

Toward the realization of thermally and ambient-stable diamond surfaces with negative electron affinity (NEA), advances in surface engineering are critical for high-performance electron-emission devices, including thermionic and field emitters, and next-generation energy converters. Here, we develop and systematically investigate a novel “molecular oxygen” oxidation method for (100)-oriented single-crystal diamond, comparing it with the benchmark UV-ozone treatment. Using the state-of-the-art surface analysis techniques, we quantify surface oxygen coverage and characterize the electronic structure following lithium deposition. The molecular oxygen treatment achieves ∼90% surface coverage and produces an NEA of −1.68 eV, outperforming UV-ozone oxidation (−1.31 eV). Although air stability is slightly limited, the NEA is fully recoverable upon reactivation (−1.56 eV). This study demonstrates that the new oxygen termination provides a practical, high-performance route to optimized NEA diamond surfaces, offering a scalable platform for next-generation electronic and energy applications.

## Graphical abstract

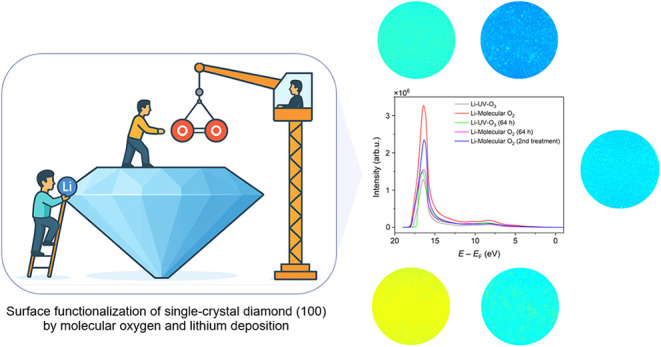

## 1 Introduction

Diamond is renowned for its exceptional mechanical strength, thermal conductivity, and unique electronic properties, making it a promising material for a variety of advanced technologies.^1,2^ Among these, efficient electron emission is particularly critical for devices such as thermionic emitters,^3–5^ secondary electron emission devices,^6^ cathode amplifiers,^7^ field-effect transistors (FETs),^8^ betavoltaic^9^ and gammavoltaic cells,^10^ and even as a solid-state source of solvated electrons.^11^ Yet, despite its intrinsic advantages, harnessing diamond in practical applications remains scientifically challenging, as it requires the controlled realization of surface terminations that enable and stabilize negative electron affinity (NEA). Notably, most conventional NEA surfaces, such as hydrogen-terminated diamond, suffer from instability under thermal or ambient conditions, with hydrogen desorption at elevated temperatures (typically ∼700 °C) leading to the loss of NEA and limiting practical use.^5,12,13^ This inherent limitation has motivated the search for alternative surface terminations that retain NEA while offering improved thermal and ambient stability. As a result, current research is increasingly focused on developing robust surface terminations, particularly those involving metal–carbon (M–C) and metal–oxygen–carbon (M–O–C) combinations, that can deliver both high NEA and long-term environmental stability.

Of these strategies, metal–oxygen (MO) terminations have shown particular promise due to their strong bonding characteristics and favorable electronic effects.^5^ Diamond surfaces with certain MO-terminations, such as LiO, MgO and ScO, have been shown to exhibit large and stable NEA.^14–16^ This stability is attributed to the strength of M–O and C–O bonds, which are generally more robust than M–C bonds.^17^ The inherent oxidative character of these terminations also lowers surface reactivity to air exposure. Furthermore, the partially ionic nature of the M–O bond enhances the surface dipole, enabling some MO-terminations to achieve NEA values even greater than those observed for H-terminated surfaces.^18,19^

A critical structural feature of these surfaces is the underlying oxygen monolayer (ML), which strongly influences the electronic structure. This layer typically consists of a mixture of ketone (C=O), ether (C–O–C), and hydroxyl (C–OH) configurations. The relative abundance of these species depends on oxidation conditions and surface coverage, with most O-terminated surfaces exhibiting a combination of these forms.^20–23^

Such oxygen terminations can be introduced *via* wet chemical treatments or gas-phase methods, using reactive atomic oxygen generated by ultraviolet (UV)-induced dissociation of ozone (O_3_) or by high-temperature cracking of oxygen (O_2_).^22–28^ While these approaches effectively functionalize the surface, the high reactivity of atomic O often leads to surface roughening.^23^ Thus, achieving a clean and controlled oxygen termination without damaging the diamond surface remains an ongoing experimental challenge. A detailed understanding of the resulting electronic, chemical, and structural properties of the oxygen ML is essential for optimizing NEA performance in diamond-based devices.

Building on the O-terminated surface, the addition of metal adsorbates can further modify the surface dipole, enhancing NEA. These dipoles are typically formed with their negative end centered on the C–O bonds and the positive end on the metal adsorbate or the overlying O layer.^29–31^ Among the metals investigated for this purpose, lithium is both well-studied and particularly promising, with experimentally demonstrated NEA values reaching as low as –2.1 eV.^14,29,32–34^ Achieving this termination requires an activation anneal, which drives structural and chemical transformations toward a more thermodynamically favorable NEA state.^14^ This process, referred to as *lithiation*, is enabled by the relatively high Li–O adsorption energy, which allows Li atoms to remain on the surface during annealing, overcoming the kinetic barrier without desorption. This strength provides another advantage, as lithiated diamond has been shown to maintain its NEA properties even at elevated temperatures.^14^

In this work, we introduce and systematically study a “molecular oxygen” oxidation technique for the single-crystal diamond (SCD) (100) system. Upon lithium activation, the method yields improved surface chemistry and electron-emission characteristics compared to the benchmark UV-ozone oxidation process, while maintaining full recoverability following ambient exposure. The new approach offers a simpler, equipment-light alternative to conventional oxidation routes, enabling broader accessibility for both research and industrial applications. Photoemission analyses using cutting-edge techniques elucidate the underlying electronic structure, establishing a clear pathway toward functionalized diamond surfaces for advanced electron-emission and energy-conversion technologies.

## 2 Experimental section

### 2.1 Sample Preparation

Pristine SCD substrates with (100) surface orientation (product code: 145-500-0549) were procured from Element Six Technologies Ltd. (Ascot, UK). The samples were initially cleaned in a boiling acid mixture for 3 h to remove any residual contamination from mechanical polishing, following the procedure described in Ref. 35 The surface topology was then characterized by atomic force microscopy (AFM), which revealed an average surface roughness (*R*_a_) of better than 2 nm (Figure S1 in Supporting Information). Since photoemission analysis requires conductive substrates and the as-received intrinsic diamond does not offer sufficient conductivity at room temperature, a boron-doped diamond (BDD) layer was grown homoepitaxially using an ASTeX-type 2.45 GHz microwave-assisted chemical vapor deposition (MW-CVD) system. The deposition was performed for 30 min using a gas mixture containing 4% CH_4_ and 5% B_2_H_6_ in H_2_, with a total gas flow of 313 standard cubic centimeters per minute (sccm), at a chamber pressure of 100 Torr and a microwave power of 1.2 kW. These growth conditions produced a ∼1 μm-thick BDD layer with a boron concentration of ≈10^20^ cm^–3^, as previously verified by secondary ion mass spectrometry (SIMS), suitable for photoemission measurements.^36^ *All* SCD (100) surfaces were then immediately H-terminated using a three-step process, as described in our prior works.^27,35,37^

The first set of freshly H-terminated samples was oxidized using the UV-ozone method, in which a mercury-vapor lamp dissociates O_3_ into reactive atomic oxygen (O_1_) and molecular O_2_ above the sample surface for 25 min.^23^ These samples were then mounted on molybdenum holders without adhesive and transferred to an ultrahigh vacuum (UHV) photoemission chamber at the Bristol NanoESCA facility and annealed at 300 °C for 1 h to remove surface contaminants.

The second set of hydrogenated samples underwent the same initial 300 °C annealing step to eliminate contaminants after introduction into the UHV chamber. The hydrogenated (100) surface was first investigated to provide a benchmark for the subsequent diamond structures (Figure S2 and Table S1). The sample was then annealed at 920 °C for 30 min to thermally desorb the H saturating dangling bonds, in preparation for termination with molecular O_2_. Following 30 min of postanneal cooling, O_2_ exposure was performed at room temperature for 20 min at a pressure of 0.6 bar using high-purity (99.999%) O_2_ gas.

Lithium was deposited from high-purity lithium wire (99.9%, Sigma-Aldrich/Merck) by thermal evaporation from a BN crucible heated by a tungsten filament. The filament temperature was held at 445 °C during deposition, which led to a deposition rate of ∼1 ML in 305 s with the sample about 200 mm from the crucible, calibrated *via* X-ray photoelectron spectroscopy (XPS). The background pressure in the deposition chamber was ∼1 × 10^–9^ mbar, rising to 8 × 10^–8^ mbar during deposition. The surface activation anneal was performed at 500 °C for 1 h.^34^

### 2.2 Photoemission Measurements

All photoemission measurements were performed using the Bristol Ultraquiet NanoESCA Laboratory (BrUNEL). Core-level XPS was performed after each treatment step using a monochromatic Al Kα source (1486.7 eV) and a Scienta-Omicron Argus analyzer positioned at 45° to the surface normal. Each measurement comprised wide-range survey scans with a pass energy of 50 eV and high-resolution scans at a pass energy of 20 eV centered on the C 1*s*, O 1*s*, and Li 1*s* core levels.

For lithiated samples, both energy-filtered photoemission electron microscopy (EF-PEEM) and region-selected ultraviolet photoelectron spectroscopy (UPS) were employed to examine the surface electronic structure. A monochromatic He I (21.2 eV) UV source (Focus GmbH) was used for both techniques. Experimental parameters, including a 37.5 μm field of view, 50 eV pass energy, 25 meV energy step, and 0.5 mm slit width, were kept constant throughout all measurements. A contrast aperture in the back focal plane was used to improve lateral resolution.

Further analysis was performed to assess the air stability of differently treated SCD(100) surfaces after 10 min and 64 h of exposure. The lithium-molecular oxygen termination was then reapplied and characterized using EF-PEEM, UPS, and XPS to evaluate the improvements achieved through repeated treatments.

## 3 Results and Discussion

### 3.1 Surface Elemental Composition

Figure S3 compares the wide-energy survey scans of the two differently oxidized samples following their respective lithiation treatments. Negligible charging effects were observed for all samples, indicating that the boron-doped layers possessed sufficient conductivity. Peak assignments in the survey scans were made using literature-reported binding energies of X-ray-excited core electrons, including 284.4 eV for BDD,^38^ 532 eV for oxygen and 55 eV for lithium.^39^

The Shirley-type background, arising from the inelastic scattering of carbon photoelectrons, is intentionally left unaccounted for in Figure S3, to emphasize its prominence.^40^ For *all* spectra containing peaks, this background was modeled in the low-energy region and subtracted prior to peak fitting. The relative percentage of chemical components at each stage of the experimental work, determined from XPS survey scans and adjusted using instrument-specific atomic sensitivity factors for each element, is summarized in Table S2.^39^

To gain insight into the chemical states resulting from the two termination approaches, narrow-energy range XPS scans of C 1*s*, O 1*s* and Li 1*s* were fitted with asymmetric Voigt function line shapes in *CasaXPS*.^41^ These empirically derived functions are a convolution of Gaussian and Lorentzian curves; the Gaussian component primarily arises due to instrumental broadening, while the Lorentzian component accounts for lifetime effects in the photoemission process.^42^ A fitting model was created for each surface treatment based on the expected chemistry and binding energy (BE) reports in the literature.^19^ This was then optimized according to the difference between the fitted model envelope and the XPS data. Model optimization was constrained to physical reasonability: the same line shape was applied to identical peaks within each binding energy region, reflecting the consistent response of the detection apparatus. In addition, the full width at half-maximum (FWHM) of constituent peaks was kept constant within each model, except where variation was justified by photoelectrons originating from distinct chemical environments.

Figure 1 presents the C 1*s* peaks for both termination methods on C(100). The large component (light blue) is assigned to bulk *sp* ^3^-hybridized carbon atoms, reported in the energy range 284.4–285.2 eV. The C 1*s* peaks pertaining to the ozone-treated diamond required a three-peak fitting model, with two additional higher BE components (Figure 1a). XPS reports on oxidized diamond surfaces^22,23^ suggest that photoelectrons excited from a C–O (ether) bonding environment have the BE shift of +1.3 eV, and that for C=O (ketone) bonds, this shift is larger at +2.8 eV. Accordingly, the two smaller peaks were assigned to C–O (green) and C=O (purple). In the C 1s model, the ratio of C–O/C=O bonding is similar for the two termination approaches, around 2:1, although this is better assessed by O 1*s* XPS scans (see later).

**Figure 1:**
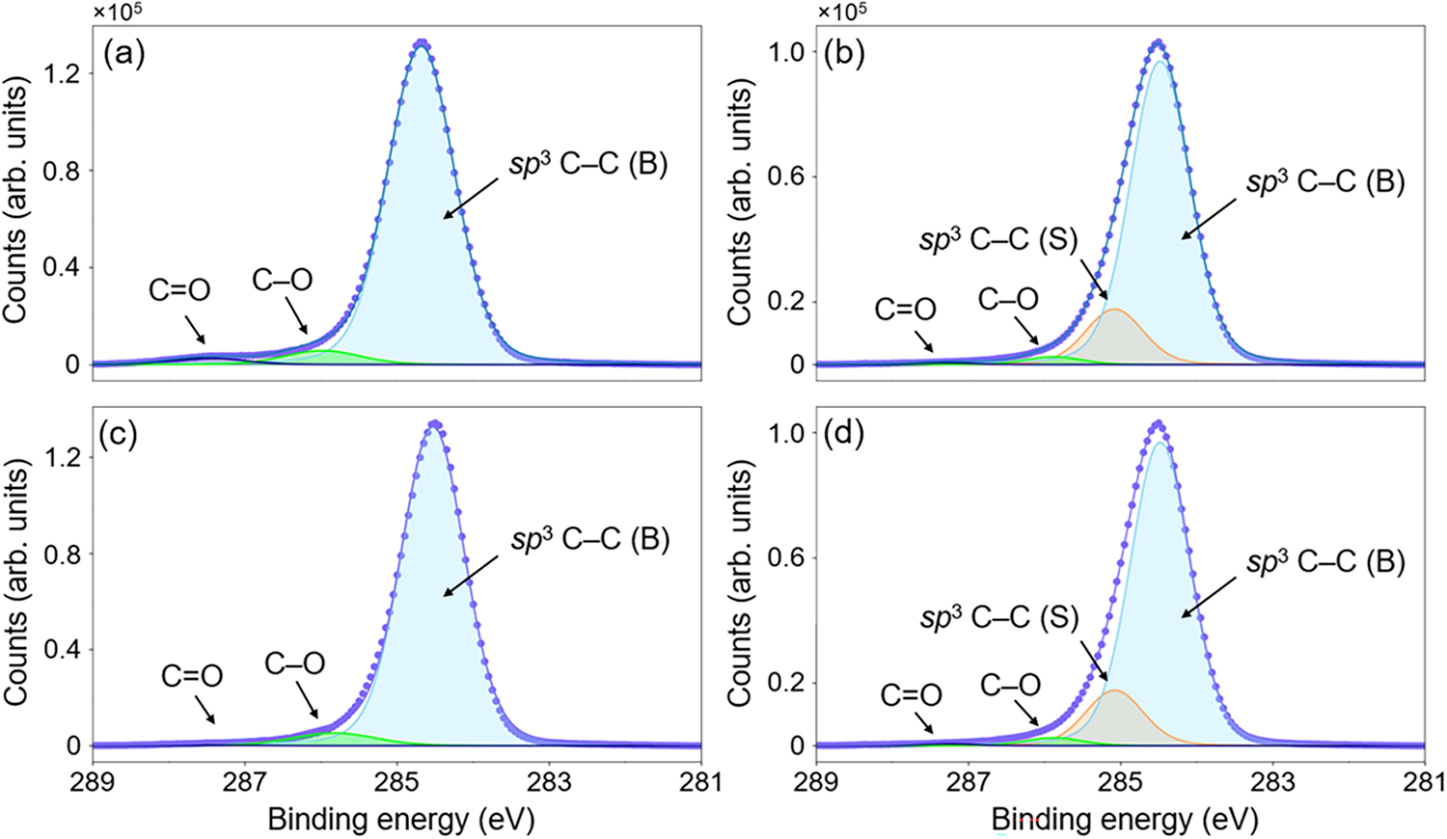
Fitted XPS C 1*s* peaks for SCD(100) surfaces after (a) UV-ozone dissociation, (b) molecular oxygen exposure, (c) lithium evaporation onto UV ozone treatment and (d) lithium evaporation onto molecular oxygen treatment. Both the raw C 1*s* XPS data and the envelope used for fitting are shown in blue.

As shown in Figure 1b, an additional component (orange) was required to obtain a reasonable fit for the molecular oxygen exposed surface, which was assigned to *sp* ^3^-hybridized carbon atoms originating from the sample surface.^23^ This is indicative of a partially oxidized diamond surface, as the BE shift from the bulk C–C photoelectron peak results from surface termination and C–H bonding, suggesting that the new (molecular oxygen) termination method developed achieves only partial surface oxidation on a C(100) surface. The requirement of an additional fourth peak in C 1*s* fitting (Figure 1b), and its similarity in BE shift to that indicative of surface C–C photoelectrons (which accounts for 10.3% of the total collected signal), further implies that this coverage was partial (Table 1). This indicates that this portion of the surface is not O-terminated by the method developed in this work.

**Table 1:** Relative Percentages and Binding Energy Position of XPS Fitting Components^a^

		UV-ozone SCD(100)		**MolO SCD(100)**	
**Treatment**	**Surface component**	**BE (eV)**	**Rel. (%)**	**BE (eV)**	**Rel. (%)**
**C 1*s***					
**Pre-lithation**	*sp* ^3^ C–C (B)	284.75	93.3	284.43	86.4
	*sp* ^3^ C–C (S)	–	–	285.03	10.3
	C–O	286.05	4.6	285.83	2.2
	C=O	287.55	2.1	287.23	1.1
**Lithiated**	*sp* ^3^ C–C (B)	284.53	94.8	284.48	82.8
	*sp* ^3^ C–C (S)			285.08	14.6
	C–O	285.83	5.0	285.88	2.1
	C=O	287.33	0.2	287.28	0.5
**O 1*s***					
**Pre-lithation**	C–O–C	532.91	11.1	533.84	2.9
	C–OH	532.05	67.6	532.31	73.3
	C=O	531.10	21.2	531.63	23.9
**Lithiated**	C–O–C	534.29	6.7	533.30	11.0
	C–OH	532.55	62.7	532.45	28.6
	C=O	531.66	11.3	531.60	19.9
	C–O–Li	530.50	19.4	530.52	13.6
	Li_2_O	–	–	529.35	27.0
**Li 1*s***					
**Lithiated**	Li^0^ (Li–Li)	55.27	19.2	54.5	32.1
	C–O–Li (Li_2_O)	–	–	55.79	67.9
	C–O–Li (LiOH)	56.52	80.8	–	–

a Obtained from C 1*s*, O 1*s* and Li 1*s* core level XPS spectra at different stages of lithiation using both UV-dissociated oxygen and molecular oxygen exposure. Note: binding energy errors in typical fits are ± 0.05–0.1 eV, therefore, relative percentages are reported to one decimal place.

In contrast, partial oxygen coverage is not observed in the XPS measurements for the comparison (UV-ozone) termination used on the same type of diamond. This is unsurprising, as O by UV-ozone dissociation is high energy, and has the ability to replace H adsorbates. However, molecular oxygen at 0.6 bar is unlikely to have energies above the adsorption energy of H to C(100),^43^ thus a partial H-termination would not be removed during the process used.^23^ Instead, it is inferred from the comparative C 1*s* fitting that the low-energy molecular oxygen saturates the remaining unterminated surface, as the energetically unfavorable unpaired carbon electrons can crack the O_2_ above them, in favor of an oxygen termination. This creates unpaired (atomic) oxygen molecules above the surface, which further terminate the surface, although evidently not in place of H-terminated regions. The H-termination resulting from the CVD growth methods was desorbed at high temperatures; for this reason, although the subsequent cooling time and presence of remnant hydrogen partial pressure in the UHV chamber make it reasonable to expect some hydrogen is readsorbed before the surface is exposed to O_2_.

To quantitatively assess the surface chemical composition of the oxygenated diamond (100) samples, the relative atomic concentrations of carbon and oxygen were extracted from the deconvoluted C 1*s* and O 1*s* XPS spectra (Figure 2a,b). This analysis provides insight into the near-surface elemental composition of the oxidized SCD surfaces and supports interpretation of their chemical and electronic properties.

**Figure 2:**
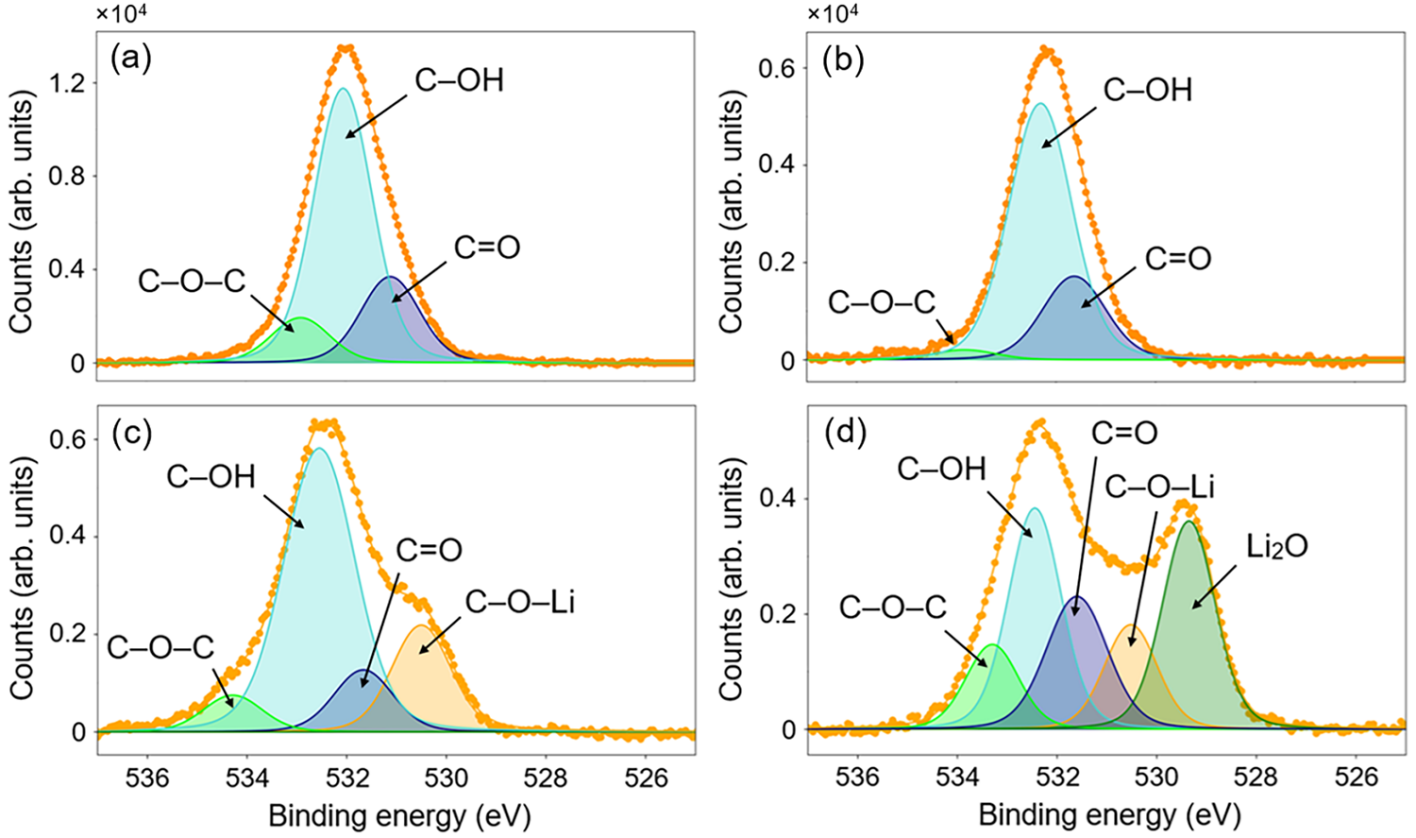
Fitted O 1s XPS measurements of SCD(100) surfaces with (a) UV-ozone dissociation, (b) molecular oxygen exposure, (c) lithium evaporation onto UV ozone treatment and (d) lithium evaporation onto a molecular oxygen treatment. The envelope used for fitting and the raw O 1*s* XPS data are shown in orange.

The oxygen atomic percentage in the oxidized surface region was calculated from the integrated peak areas of the O 1s and C 1s signals, corrected using their respective relative sensitivity factors (RSFs), according to

1 $$O\%=\frac{A\left(O\;1s\right)/RSF\left(O\;1s\right)}{\left[A\left(O\;1s\right)/RSF(O\;1s)+A\left(C\;1s\right)/RSF\left(C\;1s\right)\right]}\times 100\%$$

where *A* denotes the total fitted peak area, and the RSF ratio for O 1s to C 1s is taken as 2.93:1, based on tabulated photoionization cross sections and asymmetry parameters.^44^ This calculation assumes a uniform distribution of oxygen within the XPS probing depth. In practice, the effective oxygen percentage may be somewhat reduced due to contributions from underlying C–C bonds in the diamond lattice. To account for this effect, the analysis considers several near-surface carbon layers, consistent with the inelastic mean free path (IMFP) of photoelectrons in diamond under Al Kα excitation (∼2.7 nm).^45^

As shown in Table S2, UV-ozone oxidation produces the O-terminated diamond surface with a relative oxygen concentration of 7.49%, consistent with full oxygen coverage and in good agreement with previous reports.^22,23^ In contrast, molecular oxygen treatment yields a lower relative oxygen concentration of 6.47%, corresponding to an effective oxygen surface coverage of ∼90%. This difference reflects the distinct oxidation mechanisms associated with the two treatments, as discussed above.

Based on the C 1*s* model, the energy and distribution of photoelectrons from both oxidized surfaces remained largely unchanged because of the lithiation process; thus, an inspection of the O 1s peak was completed to obtain a better comparison. XPS measurements of the two differently terminated surfaces in the oxygen photoelectron BE region are shown in Figure 2a,b. Fitting these was initially completed with a three-peak model, in line with the C 1*s* model, with two peaks expected to result, centered around the BEs of ether (C–O–C) at 532.7 eV (green) and ketone (C=O) at 531.1 eV (blue). The third and most prominent peak is assigned to C–OH bonding environments, for which the reported central BE is around 532 eV.^21–23^

In lieu of the C 1*s* peak structure change following lithium evaporation, the O 1s and Li 1*s* peaks are used to compare the lithium uptake between termination methods. Fitting of the O 1*s* peaks indicates both surfaces have similar chemistry prior to Li deposition: the concentration of C–OH complexes is similar, and, in the absence of an O=O peak, negligible O_2_ is present on both surfaces. However, the primary difference in the O photoemission is that the molecular oxygen-treated surface exhibits a higher proportion of ketone bonding (C=O/C–O–C = 8.2) compared with the UV-treated sample (C=O/C–O–C = 1.9) (Table 1). After Li deposition, this ratio changes only slightly for both oxidized surfaces, while the C–OH concentration decreases markedly, indicating that Li preferentially bonds to C–OH groups.

In addition, following Li addition, both O-terminated surfaces developed additional low-BE features, requiring updated fitting models: a four-peak model for the UV-ozone-treated surface and a five-peak model for the molecular oxygen-treated surface (Figure 2c,d). The fitting model for Li on the ozone-treated surface was less complex, with only one additional peak corresponding to C–O–Li photoelectrons, whereas the molecular oxygen-exposed surface exhibited two distinct bonding features of C–O–Li and Li_2_O. These features are consistent with C–O–Li (530.4 eV, orange) and Li_2_O (∼528.5–529.5 eV, dark green) environments, both of which cause a negative BE shift.^14,33,46^

The Li core-level photoelectron peak is typically centered at 54.6–55.5 eV.^14,33,46^ The XPS spectra in Figure 3 cover this range and reveal additional structure at higher BEs. For all samples, the Li 1*s* spectra were fitted using a two-peak model: a lower BE peak at 54.5–55.3 eV, assigned to metallic Li (purple), which manifests as Li–Li dendritic aggregates, and a higher BE peak was attributed to C–O–Li species with ionic character (*i.e*. Li_2_O and LiOH), consistent with the O 1s scan fitting. Reported Li 1*s* photoelectron BEs for Li_2_O and LiOH are closely spaced, typically ranging from 55.5 to 57 eV.^46^ Furthermore, due to the weak XPS response to lithium core-level photoelectrons, the Li spectra exhibit lower intensity than the C 1*s* and O 1*s* peaks. It is therefore reasonable to group similar BE photoelectron signatures for comparison, as a more complex fitting model could be misleading. The BE values for each curve fitted to the XPS measurements during the lithiation comparison, along with the relative percentage of chemical components obtained from the peak fitting, are summarized in Table 1.

**Figure 3:**
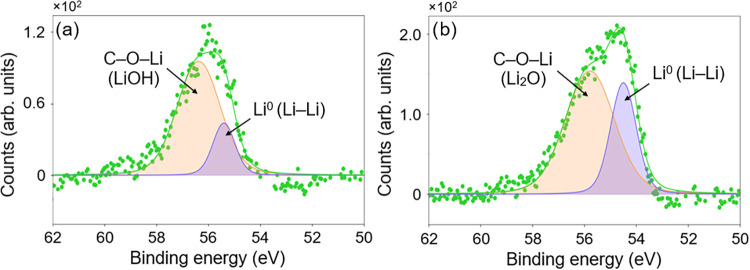
Fitted core-level spectra over the Li binding energy range for (a) lithiated, UV ozone-terminated SCD(100) and (b) lithiated, molecular oxygen-terminated SCD (100).

Lithium adsorption on the molecular oxygen-treated sample was greater, while the oxygen–carbon chemistry remained similar for both surfaces after lithium deposition (Figure 2a,b). This is evidenced in the comparative normalized Li 1s peaks intensity and the O 1s peak fitting, as the molecular oxygen peak gained increased structure at lower BE, indicative of lithium–oxygen bonding. While this alone does not conclusively demonstrate a larger number of NEA-inducing Li–O dipoles on the molecular oxygen surface, the presence of Li–O-related components, including Li_2_O-like species associated with surface-bonded C–O–Li configurations, suggests an increased surface dipole that may contribute to more NEA. This is further supported by a higher relative concentration of C–O–Li observed for the molecular O-terminated surface compared to the UV-ozone one, consistent with the larger NEA recorded (Figure 2c,d). A detailed analysis of the surface electronic structure changes, specifically NEA and work function (WF), will follow in section 3.2.

LiO-terminated diamond has previously been reported to be stable in ambient conditions.^14,33^ To test this claim, and to assess whether it also applies to the Li–O-diamond surface oxidized using the new method developed in this study (*i.e*., molecular oxygen), both LiO-terminations were further analyzed after exposure to the atmosphere (30–40% humidity, 18.8–21.9 ^◦^C) for 10 min, followed by 64 h.

### 3.2 Surface Electronic Structure

To elucidate the surface electronic structure of LiO-terminated diamond (100) prepared *via* two different oxidation methods, ultraviolet photoelectron spectroscopy (UPS) and energy-filtered photoemission electron microscopy (EF-PEEM) were performed after each step of the sample preparation process. As a benchmark for the LiO-terminated diamond surfaces, the oxidized SCD (100) samples were first investigated, and the corresponding results are provided in Figure S4 and Table S3.

Figure 4 presents local WF maps produced by EF-PEEM measurements of the differently treated samples. Although the Li-adsorbed UV-ozone oxidized sample exhibits a uniform surface over a 37.5 μm area, the molecular oxygen-treated Li–O–C(100) sample shows greater variation in the surface electronic structure, with a local WF of 3.21 eV (Figure 4a,b). The appearance of patches across the surface is likely attributable to partial oxygen coverage and is also consistent with the presence of aggregated metallic Li domains.

**Figure 4:**
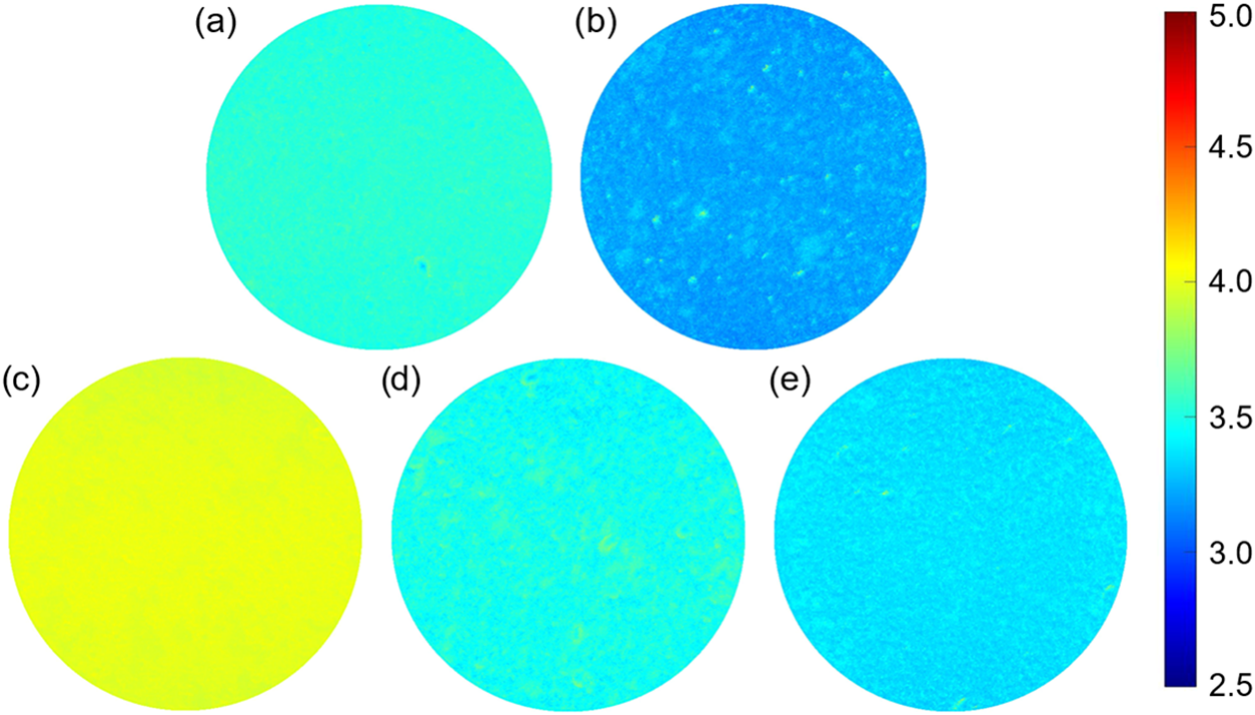
Color-coded local WF maps of the surface of (a) lithiated, UV-ozone terminated C(100), (b) lithiated, molecular oxygen terminated C(100), (c) lithiated, UV-ozone terminated C(100) exposed to atmosphere for 64 h, (d) lithiated, molecular oxygen terminated C(100) exposed to atmosphere for 64 h and (e) the sample in (d) after being treated for a second time with the same molecular oxygen and lithium evaporation process. The coloring indicates the local WF of that area of the sample in eV, with a scale represented by the adjacent axis. The field of view for each map is 37.5 μm.

To further probe these spatial variations in electronic structure, region-selected UPS measurements were performed over the same area for *all* lithiated samples Figure 5. All UPS spectra are normalized to the “knee feature”,^47^ which corresponds to the minimum energy required to create an electron–hole pair and is therefore at a suitably higher energy than the vacuum level and conduction band minimum (CBM) for accurate quantification.^48^ Notably, the characteristic NEA peak appears in *all* UPS spectra, albeit with varying intensities. This variation is reflected in the numerical data calculated from the electronic structure measurements, which quantitatively capture differences in NEA values across the lithiated samples. To determine these values, electronic structure parameters were extracted using multiple methods. From the EF-PEEM maps, surface-averaged local WF values were determined *via* pixel-by-pixel selection within the defined area of interest.^16,35,49^ Alternative WF values, along with additional electronic structure information, were obtained from UPS spectra recorded over the same areas. In light of this, a method developed by Maier et al.^19^ was used to interpret the valence band maximum (VBM) and CBM, as expressed in eq 2 for each sample:

2 $$E_{\mathrm{CBM}}-{(E}_{F})=\mathrm{hv}-{(E}_{F}-E_{\mathrm{VBM}})-E_{g}$$

where *E*_VBM_ and *E*_CBM_ denote the energies of the VBM and CBM, respectively, *E*_F_ is the Fermi energy, *hv* is the energy of the He (I) photon source (21.22 eV), and *E*_g_ is the experimental band gap of diamond (5.47 eV). This analysis was supported by C 1s core-level XPS scans collected prior to UPS measurements, where the shift in the bulk C–C sp^3^ BE from 283.9 ± 0.1 eV was taken to correspond to *E*_F_ – *E*_VBM_.^50^ This value was then used to calculate EA values (χ) from the following equation:

3 $$\chi =\phi +{(E}_{F}-E_{\mathrm{VBM}})-E_{g}$$

where ϕ represents the WF. For the calculation of NEA values *via* eq 3 (Table 2), electronic energy levels derived from XPS and EF-PEEM analyses were used, while UPS data provided additional support for this interpretation (Table S4). While the different approaches used to determine the defining energies produce varying absolute values, they consistently show that the NEA magnitude of the Li-on-molecular-oxygen-terminated sample is greater than that of the Li-adsorbed UV-ozone-treated surface. This trend aligns with EF-PEEM measurements of the same areas, which reveal lower WF surface coverage for Li on the molecular oxygen-treated sample.

**Figure 5:**
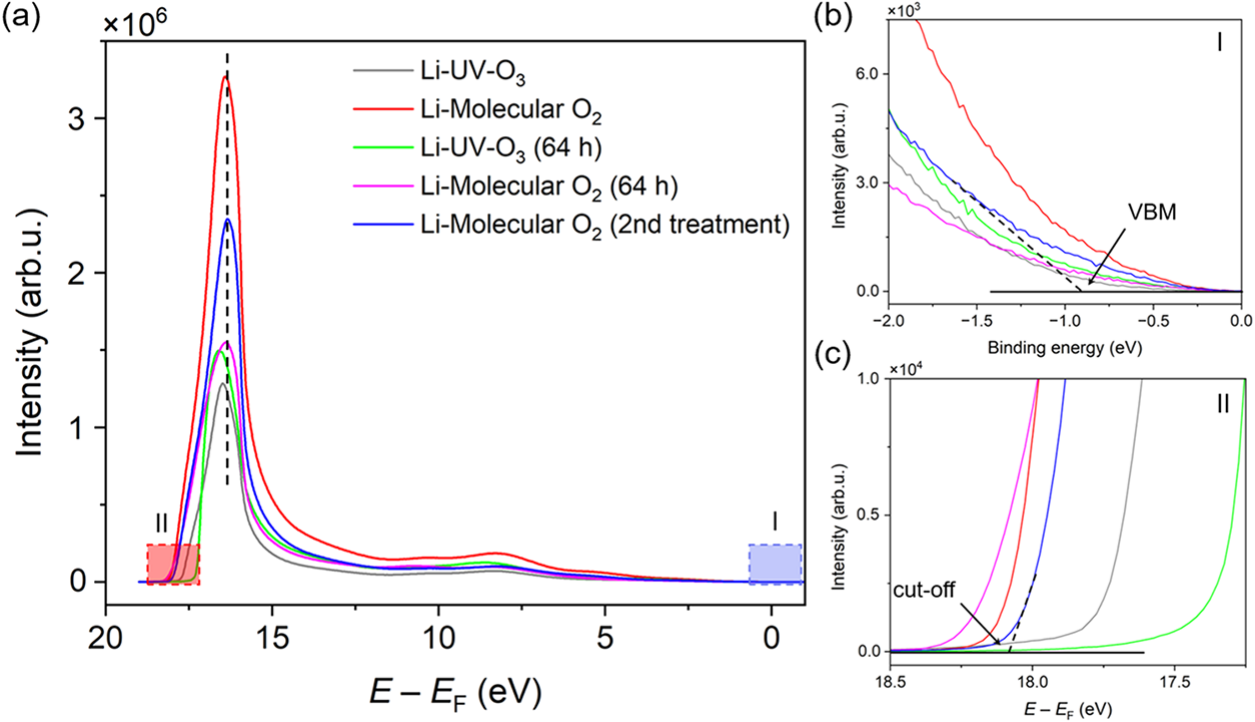
(a) UPS spectra of differently treated diamond surfaces, presented over the full photoelectron binding energy range measured. For the Li on molecular oxygen-terminated SCD(100) sample after a second treatment (blue), the CBM position (relative to photon energy) found at the NEA peak maximum is presented (black dashed line). The same spectra are presented below, cropped to (b) region I: the low binding energy region that encodes information on the VBM position relative to *E*_F_, and (c) region II: the secondary electron cutoff region, from which the work function value was found.

**Table 2:** Electronic Structure Energy Values of the LiO-terminated Diamond (100) Ssurface, Obtained from XPS and EF-PEEM Measurements

	***E***_**F**_ **–** ***E***_**VBM**_ **(eV)**	**ϕ** **(eV)**	***χ***
**Treatment**	**XPS**	**XPS+PEEM**	**XPS+PEEM**
Li-ozone	0.63 ± 0.1	3.53 ± 0.03	–1.31 ± 0.16
Li-MolO	0.58 ± 0.1	3.21 ± 0.06	–1.68 ± 0.18
Li-ozone (68-h air exposure)	0.83 ± 0.1	3.99 ± 0.02	–0.65 ± 0.14
Li-MolO (68-h air exposure)	1.14 ± 0.1	3.47 ± 0.04	–0.86 ± 0.18
Li-MolO (second treatment)	0.55 ± 0.1	3.36 ± 0.04	–1.56 ± 0.16

For UPS extrapolations, the error range was estimated using two alternative limits for extrapolating the linear decline (Figure 5). In contrast, for the local WF values obtained from PEEM, the error is substantially smaller and corresponds to the standard deviation within the selected averaging region. The CBM energy values extracted from the NEA peak carry an uncertainty equal to the peak’s fwhm, while the error associated with the XPS-measured VBM is given in the details of the method.^19^ With this in mind, the NEA value calculations presented in Table 2 use a combination of PEEM-derived local WF values and Maier’s method on C 1*s* XPS measurements, as this approach is considered the most accurate. The main limitation of UPS is that the VBM signal is weak and characterized by a shallow gradient, which introduces considerable uncertainty in determining its intersection with the *y*-axis. Maier’s method helps to mitigate these inaccuracies by reducing the dependence on UPS *E*_F_ calibration; however, because it relies heavily on the accuracy of XPS fitting, repeating the UPS measurements after calibration would still be valuable for confirmation. This advantage is reinforced by the fact that surface-averaged local WF values obtained from PEEM exhibit a smaller error range than those derived from UPS extrapolations, making the PEEM-based data more reliable for predicting accurate NEA values.

The resulting electronic structures, determined using a combination of XPS, UPS and PEEM, are depicted in Figure 6. All *E*_F_ – *E*_VBM_ values obtained in this work are significantly larger than expected for the bulk Fermi level position (∼0.1 eV) in heavily BDD (Table 2); this deviation is attributed to strong surface Fermi-level pinning and associated band bending induced by surface states, rather than to errors in the UPS or XPS measurements. Other approaches for determining NEA magnitudes, applying UPS, PEEM + UPS, and XPS + UPS, are summarized in Table S5.

**Figure 6:**
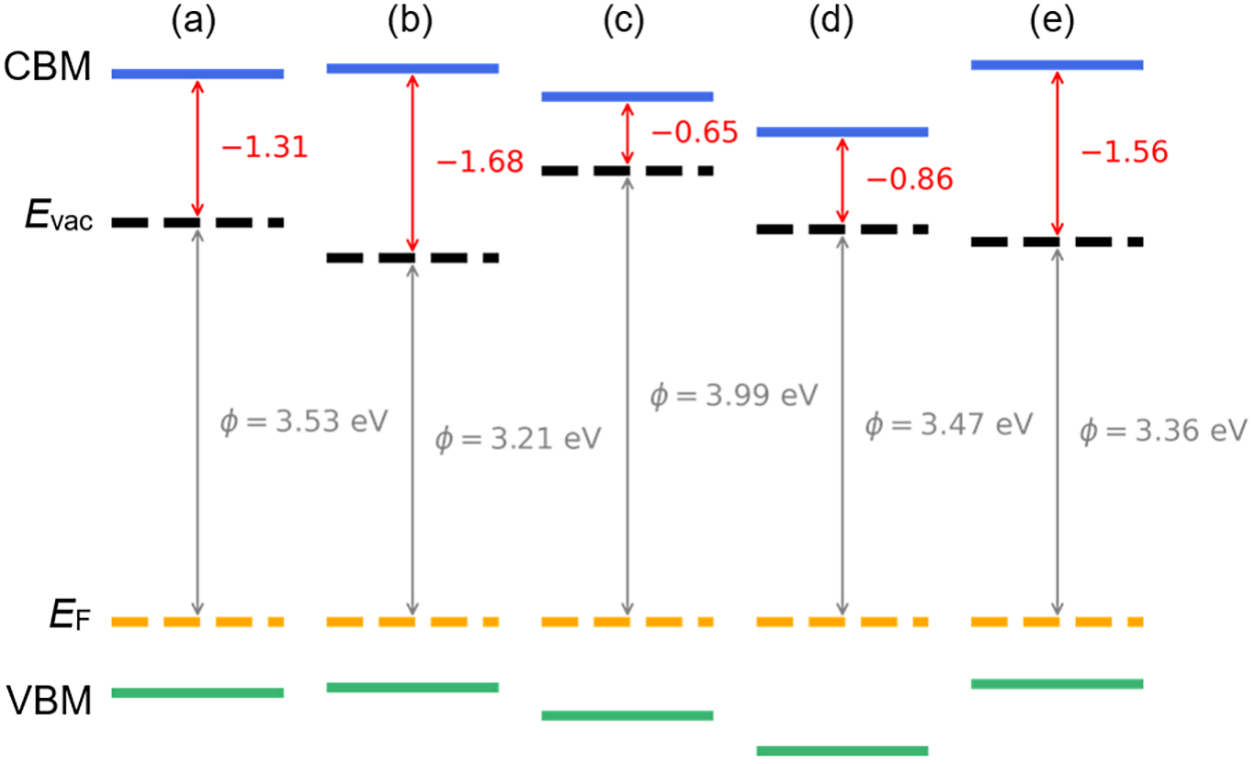
Band structure diagrams obtained from XPS, UPS and EF-PEEM for the Li-adsorbed (a) UV-ozone-treated C(100), (b) Molecular oxygen-treated C(100). (c) and (d) Postair exposure measurements for UV-ozone treated C(100) and molecular oxygen-treated C(100), respectively. (e) Molecular oxygen treated C(100) after 64 h of exposure to air and a second treatment.

### 3.3 Air Stability and Recoverability

In EF-PEEM and region-selected UPS measurements, the NEA decreased for both oxygen termination methods, as shown in Figures 4 and 5. This concurs with O 1*s* XPS fitting, where after the 10 min exposure, the low BE (lithium–oxygen) structure for both samples was diminished significantly (Figure 7a,c). This structural change is most noticeable for the molecular oxygen sample, which, after exposure, takes a shape similar to before Li was deposited. If the molecular oxygen termination is partially oxidized and/or H-terminated, this surface would likely be less stable in the atmosphere: the incomplete coverage would leave the surface susceptible to alien adsorbates, and H is known to be unstable outside of UHV.^51,52^ Future work using low-energy electron diffraction (LEED) could quantify the partial oxidation of the molecular oxygen treatment, because while the Li–O complex does not reconstruct the C(100) surface, H-terminated regions would be (2 × 1) reconstructed and could be identified. The LiO-terminated surface has a higher BE than H-termination, so this could also be investigated with high-temperature anneal XPS measurements.

**Figure 7:**
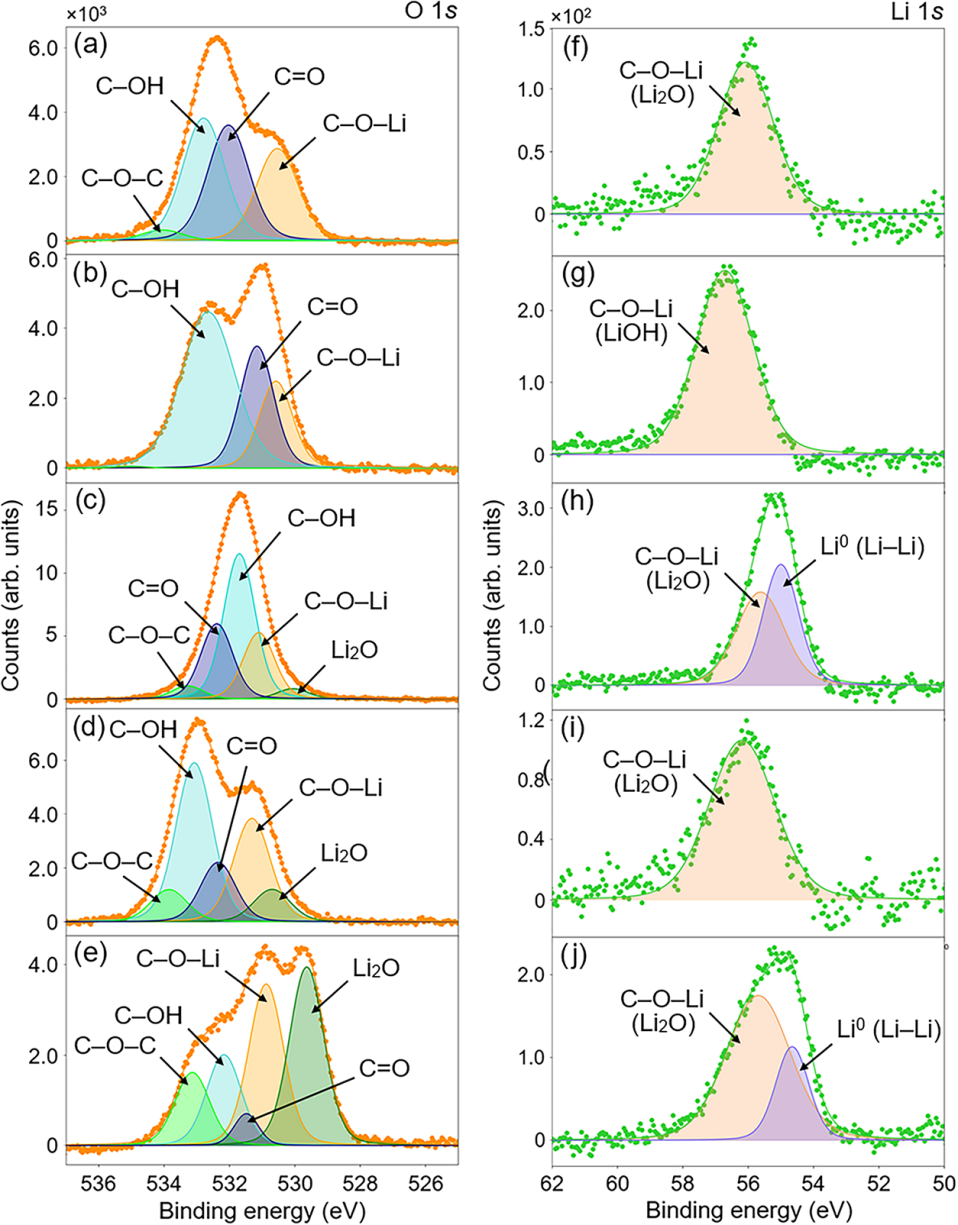
Core-level XPS spectra of O 1*s* and Li 1*s* for two LiO-terminated surfaces. (a, b) O 1*s* and (f, g) Li 1*s* fitted peaks of lithiated UV-ozone C(100) after 10 min and 64 h of air exposure. (c, d, e) O 1*s* and (h, i, j) Li 1*s* fitted peaks of lithiated molecular oxygen C(100) following 10 min and 64 h of exposure to ambient conditions, as well as after the second treatment, respectively.

After 10 min of atmospheric exposure, the samples were only annealed for 10 min before XPS measurements. Therefore, the change in structure observed is likely to be the result of surface contaminants, so this is not representative of the effect on termination structure. After 64 h of exposure, the samples were annealed for an hour, and in the subsequent XPS scan, much of the structure seen before exposure is regained. These measurements were followed by EF-PEEM and UPS measurements, and therefore, are more valuable for air stability analysis (see Figures 4c,d and 5). Furthermore, both exposure measurements were made on the same samples, so the second measurement represents the impact of both exposures. Information on air stability after 10 min of air exposure is also less valuable from a device perspective, where an NEA surface that is stable over a much longer period is desired. From this viewpoint, both the lithium molecular oxygen and the UV-ozone treatments are unappealing, as, contrary to the literature, the NEA they produced was degraded by long-term air exposure (*i.e.*, 64 h). With similar reasoning to the lower magnitude NEA, this could be due to the activation anneal temperature, although more work is needed to determine why both surfaces were not stable to the atmosphere.

These electronic structure changes are also reflected in the XPS fitting of Li photoelectron peaks, as shown in Figures 3 and 7f,g,h,i,j, although this is not initially apparent. These peaks were fitted with metal and ionic components (Li_2_O and LiOH), the latter of which is symptomatic of the NEA inducing dipoles on LiO-terminated diamond.^14^ Fitting XPS scans of the surfaces with the same model after 64 h of exposure to the atmosphere indicates that only ionic compounds remained. This suggests that the surface has a termination that would better induce NEA; however based on the UV photoemission measurements (Figure S5), this component is best attributed to lithium–oxygen complexes (with some Li_2_O-like character) that are unlikely to remain directly bonded to the diamond surface in a configuration that forms an outwardly electropositive dipole. This hypothesis also conforms to the XPS O 1s scans, where air exposure is concomitant with a significant decrease in intensity of Li_2_O components, from 27 to 8.5%, for the molecular oxygen-treated sample (see Tables 1 and 3). However, the ratio of Li on the surface, quantified by survey scans (Table S2), indicates only a slight change in the total Li content following the long air exposure (*i.e.*, 64 h) for both surfaces. Based on these results, the NEA-diminishing changes that result from atmosphere exposure are likely structural, *i.e.*, dipole destroying, rather than a consequence of large amounts of Li desorbing from the sample surface.

**Table 3:** Relative Component Percentages of LiO-terminated C(100) Surfaces^a^

		**UV ozone C(100)**		**MolO C(100)**	
**Treatment**	**Surface component**	**BE (eV)**	**Rel**. **(%)**	**BE (eV)**	**Rel**. **(%)**
**C 1*s***					
**10 min air exposure**	*sp* ^3^ C–C (B)	284.61	94.3	284.34	81.6
	*sp* ^3^ C–C (S)	–	–	284.94	15.7
	C–C	285.91	4.9	285.74	2.3
	C=C	287.41	0.8	287.14	0.4
64-h air exposure	*sp* ^3^ C–C (B)	284.73	96.8	285.04	96.0
	*sp* ^3^ C–C (S)	286.03	3.0	285.94	1.4
	C–C	287.53	0.2	286.44	0.4
	C=C	–	–	287.84	2.2
**O 1*s***					
**10 min air exposure**	C–O–C	534.05	3.1	533.28	4.2
	C–OH	532.79	36.3	532.39	46.8
	C=O	532.03	33.7	531.70	24.4
	C–O–Li	530.73	26.9	531.10	21.4
	Li_2_O	–	–	530.06	3.3
64-h air exposure	C–O–C	535.02	0.4	533.85	8.4
	C–OH	532.66	54.3	533.08	41.5
	C=O	531.16	26.4	532.35	14.7
	C–O–Li	530.78	18.9	531.32	27.0
	Li_2_O	–	–	530.70	8.5
**Li 1*s***					
**10 min air exposure**	Li^0^ (Li–Li)	–	–	54.65	25.1
	C–O–Li (Li_2_O)	56.08	100	55.69	74.9
64-h air exposure	C–O–Li (Li_2_O)	–	–	56.12	100
	C–O–Li (LiOH)	56.72	100	–	–
**2**nd **treatment**	Li^0^ (Li–Li)	–	–	54.5	32.1
	C–O–Li (Li_2_O)	–	–	55.79	67.9

a Obtained from binding energy shift fitting models of C 1*s*, O 1*s* and Li 1*s* core level XPS spectra for the two different oxidation methods, following 10 min and 64 h of atmosphere exposure and a second molecular oxygen and lithium treatment. Note: binding energy (BE) errors in typical fits are ± 0.05–0.1 eV, therefore, relative percentages are reported to one decimal place.

Treating the lithium and molecular oxygen exposed C(100) for a second time, intended to investigate if the NEA magnitude could be enhanced by further treatments, which could saturate any unterminated regions and potentially increase the size of the dipoles already formed. In light of the degradation observed after air exposure, this is instead a measurement of the recoverability: can further treatments regain the NEA of the termination before it was exposed to the atmosphere? Further measurements carried out on the initial intention (without air exposure) would be valuable to understand the lithium and molecular oxygen-terminated surface. Nonetheless, the recoverability of the termination appears excellent, with the fitted peaks regaining a structure and relative atomic concentration similar to the first deposition, and the NEA returning to just 0.19 eV lower in magnitude, as observed in EF-PEEM and UPS data (Figures 4e and 5).

## 4 Conclusions

We have developed and systematically characterized a novel molecular oxygen oxidation method for SCD(100), achieving ∼90% surface coverage and higher lithium uptake than the benchmark UV-ozone technique, without the need for specialized equipment. Surface analysis *via* XPS, EF-PEEM, and UPS confirms that this termination supports a significantly larger NEA (−1.68 eV) than UV-ozone oxidation (−1.31 eV), attributed to increased lithium incorporation and distinct oxygen bonding environments. Although air stability remains a limitation, likely arising from the lithium activation anneal, the NEA is readily recoverable (−1.56 eV) following reactivation. The simplicity, effectiveness, and strong electron-emission characteristics of this new molecular oxygen method make it a promising candidate for NEA-dependent technologies that use synthetic diamond, including thermionic energy converters, high-gain secondary electron emission devices, β voltaic nuclear batteries, and advanced radiation detectors for fusion applications.

## Supplementary Material



## Notes

AFM surface morphology and roughness analysis, UPS, XPS, and EF-PEEM measurements of electronic structure parameters, surface composition analysis during lithiation and air exposure, comparison of NEA values obtained by multiple spectroscopic methods, and additional UPS measurements following repeated treatments (PDF)
