# Supplementary material for: Achieving a Large Net “Negative Electron Affinity” on Diamond (100) via Molecular Oxygen and Lithium Functionalization
Source: ACS Appl Mater Interfaces. 2026 Jan 27;18(5):9032–42. doi: 10.1021/acsami.5c20029 (PMC12903099; doi:10.1021/acsami.5c20029)
Supplement: Supplementary file 1 [file am5c20029_si_001.pdf]

Supporting Information for

Achieving a Large Net 'Negative Electron Affinity' on Diamond (100) *via* Molecular Oxygen and Lithium Functionalization

Ramiz Zulkharnay[a*], William Greenwood,[b] Adam Wood[b], Jude Laverock[a] and Neil A. Fox[a,b]

[a] *School of Chemistry, University of Bristol, Cantock's Close, Bristol, BS8 1TS, UK*

[b] *School of Physics, H.H. Wills Physics Laboratory, University of Bristol, Tyndall Avenue, Bristol, BS8 1TL, UK*

*Corresponding author: E-mail: ramiz.zulkharnay@bristol.ac.uk

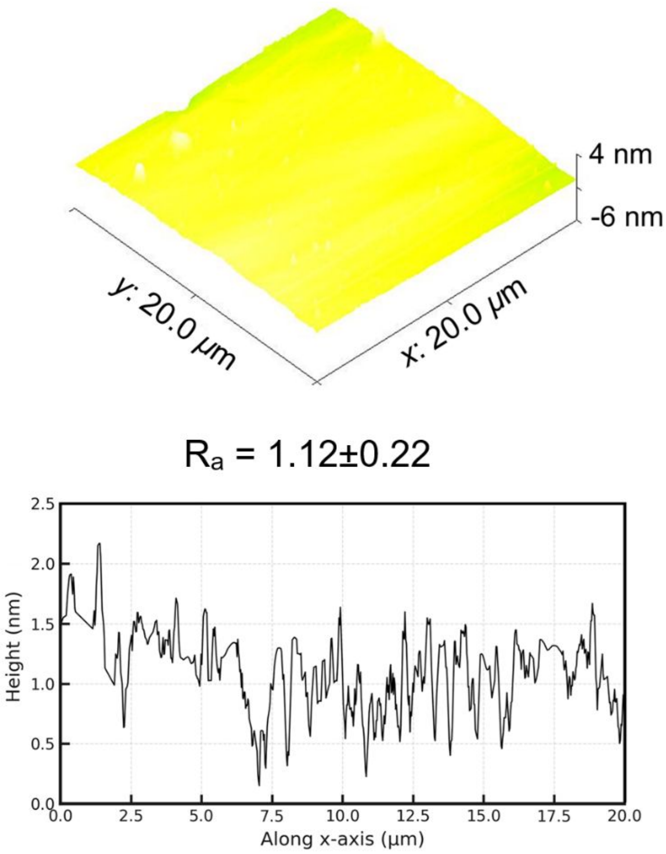

Figure S1. Atomic Force Microscopy (AFM) 3D map with corresponding surface roughness plot for as-received SCD(100) sample, after acid washing procedures.

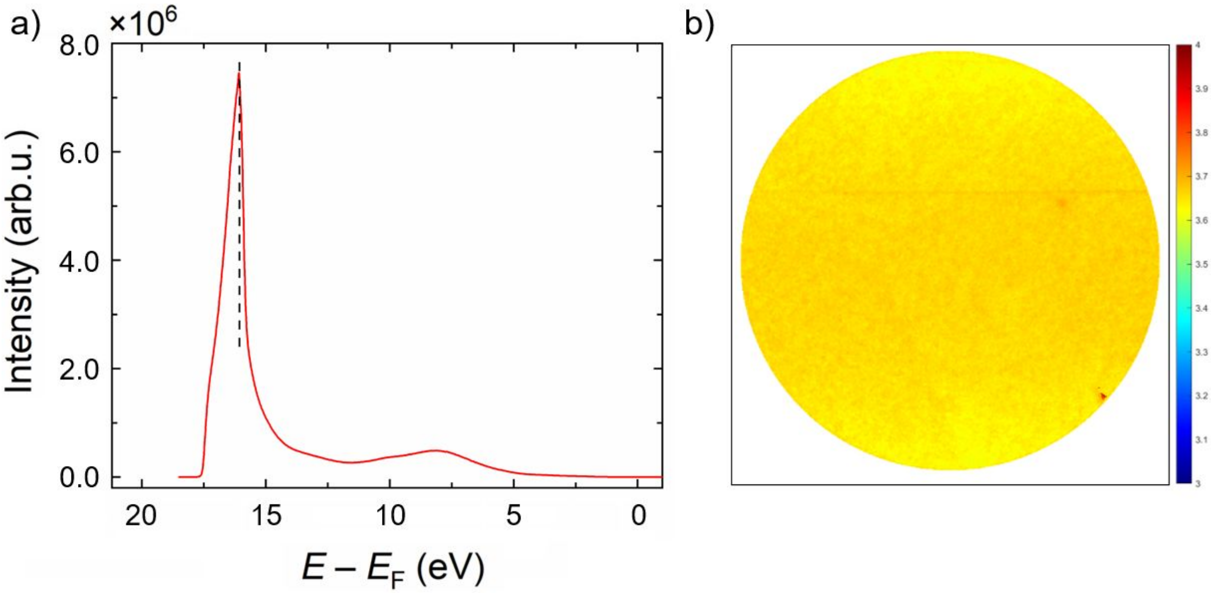

Figure S2. (a) Region-selected UPS spectra and (b) color-coded local WF map of the hydrogenated SCD(100) sample, taken with an energy resolution of 0.14 eV and excitation energy of 21.22 eV. For the local WF map, the field of view is 37.5 μm.

Table S1. Electronic structure energy values of the hydrogenated SCD(100) sample obtained using region-selected UPS.

**Treatment**	$E_F - E_{VBM}$ **(eV)**	ϕ **(eV)**	χ
H-terminated SCD(100)	0.75 ± 0.05	3.67 ± 0.12	-1.05 ± 0.55

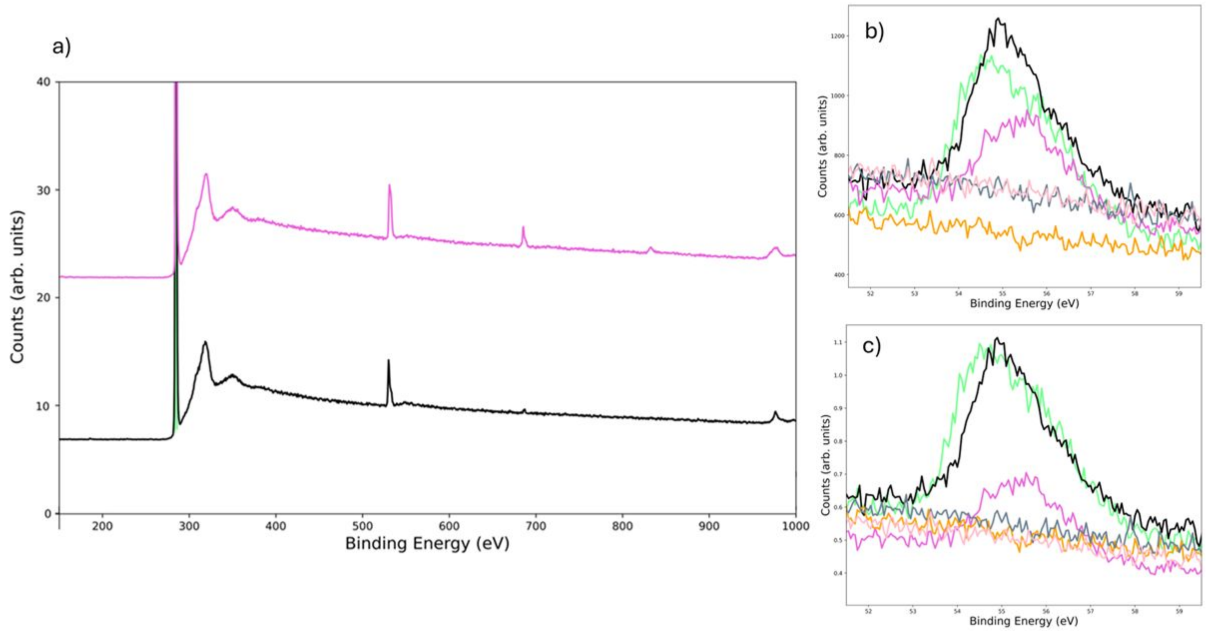

Figure S3. a) XPS survey of lithiated diamond samples using: UV-ozone termination on C(100) (purple) and molecular oxygen on C(100) (black). As expected for the oxidized diamond, both spectra are dominated by the intensity of the C 1*s* peak, with a less intense O 1*s* peak. Other notable peaks can be seen at the expected binding energy of fluorine (a common UHV contaminant) and from Auger electron emission from carbon and oxygen. Narrow energy XPS scans over the energy range expected for lithium core electrons during the same measurements (plotted in the same color), are presented as b) unnormalized and c) normalized using the carbon peak intensity for each measurement. The same measurement was repeated for the three samples (green, black and orange, respectively) before lithiation for comparison of uptake.

Table S2. Survey scan area quantification of the relative atomic concentration of carbon, oxygen, lithium and fluorine peaks at all stages of the experimental process. Note: F is a common contaminant in UHV and transportation, and likely was removed during pre-measurement annealing.

**Termination**	**Treatment stages**	**Relative atomic concentration (%)**			
		**C**	**O**	**Li**	**F**
Hydrogen	-	98.28	1.71	0.00	0.01
UV Ozone	-	92.51	7.49	0.00	0.00
	Post Li Deposition	88.98	2.92	7.64	0.47
	Pre-Air Exposure	85.41	3.11	9.93	1.56
	10 Min Air Exposure	87.70	3.25	7.98	1.07
	68-Hour Air Exposure	86.12	3.07	10.75	0.06
	-	99.07	0.91	0.00	0.02
Hydrogen	-	98.33	1.67	0.00	0.00
Molecular Oxygen	-	93.23	6.73	0.00	0.04
	Post Li Deposition	81.78	4.06	13.68	0.48
	Pre-Air Exposure	81.51	4.06	12.83	1.61
	10 Min Air Exposure	77.39	7.47	13.68	1.46
	68-Hour Air Exposure	80.80	4.84	12.75	1.61
	2nd O + Li Treatment	80.33	5.26	14.14	0.27

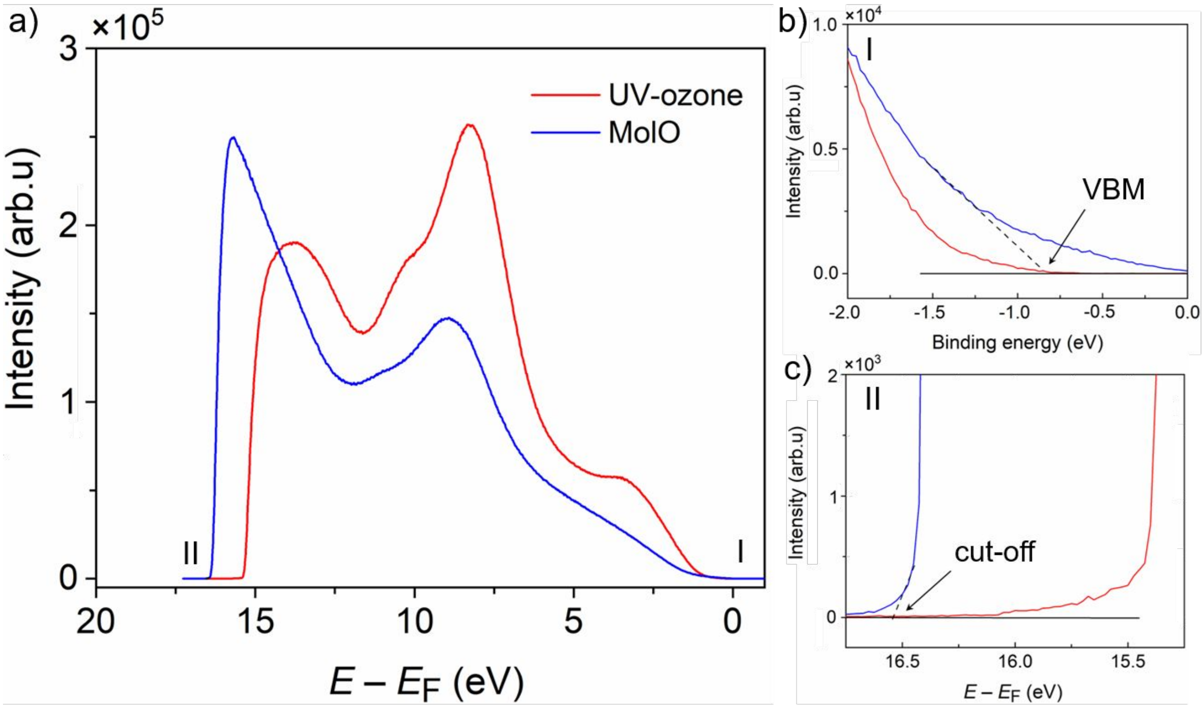

Figure S4. Region-selected UPS spectra of two O-terminated diamond (100) surfaces: a) full-range spectra with two regions of interest, I and II. (b) Magnified view of region I showing the valence band maximum (VBM) position relative to the Fermi level, and (c) enlarged view of region II showing the secondary electron cut-off.

Table S3. Electronic energy values of O-terminated diamond (100) surfaces measured using region-selected UPS.

**Treatment**	$E_F - E_{VBM}$ **(eV)**	ϕ **(eV)**	χ
UV-Ozone	1.31 ± 0.05	5.82 ± 0.09	1.66 ± 0.58
MoIO	0.88 ± 0.11	4.72 ± 0.15	0.13 ± 0.58

Table S4. Electronic structure energy values of the LiO-terminated diamond (100) surface obtained using region-selected UPS.

Treatment	$E_F - E_{VBM}$ (eV)	ϕ (eV)	E_{CBM} (eV)
Li-Ozone	1.05 ± 0.06	3.47 ± 0.11	4.73 ± 0.58
Li-MolO	0.91 ± 0.11	3.18 ± 0.17	4.85 ± 0.58
Li-Ozone (68-h air exposure)	0.83 ± 0.18	3.93 ± 0.03	4.58 ± 0.58
Li-MolO (68-h air exposure)	1.00 ± 0.11	3.13 ± 0.13	4.83 ± 0.58
Li-MolO (2nd treatment)	0.86 ± 0.15	3.25 ± 0.12	4.78 ± 0.58

Table S5. The NEA values found in this work using a) UPS, b) PEEM and UPS, c) XPS and UPS, d) XPS and PEEM.

Diamond	Treatment	a)	b)	c)	d)
C(100)	Li-MolO	-1.38 ± 0.31	-1.35 ± 0.18	-1.71 ± 0.30	-1.68 ± 0.18
C(100)	Li-Ozone	-0.95 ± 0.19	-0.89 ± 0.11	-1.37 ± 0.23	-1.31 ± 0.16
C(100)	Li-MolO (68 Hour Air Exposure)	-1.33 ± 0.25	-1.00 ± 0.19	-1.20 ± 0.25	-0.86 ± 0.18
C(100)	Li-Ozone (68 Hour Air Exposure)	-0.46 ± 0.23	-0.41 ± 0.22	-0.71 ± 0.16	-0.65 ± 0.14
C(100)	Li-MolO (2nd Treatment)	-1.36 ± 0.29	-1.26 ± 0.21	-1.67 ± 0.24	-1.56 ± 0.16

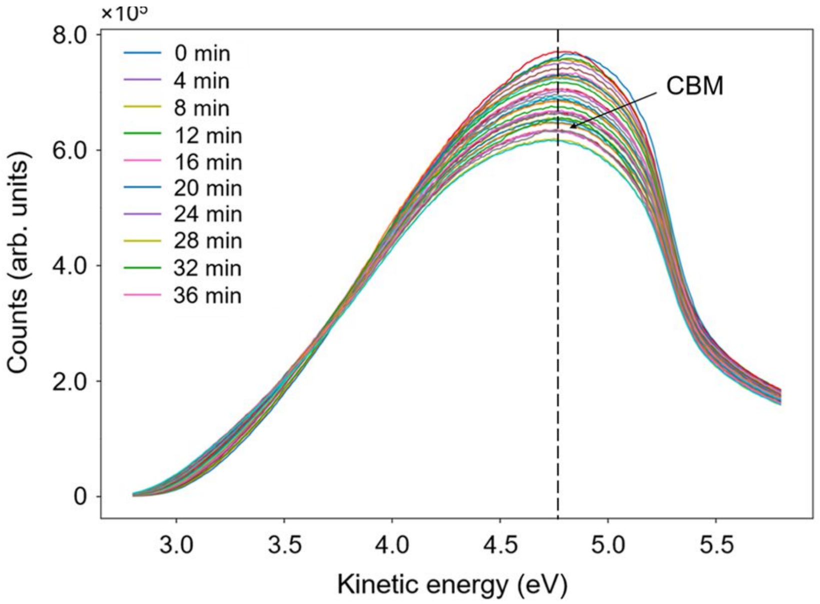

Figure S5. UPS scans of air-exposed Li-O-C(100) following a second molecular oxygen and lithium treatment, each scan is presented individually. A decrease in NEA peak height can be observed throughout the measurement, a feature which is not present in the other UPS measurements in this work. The black dashed line indicates the CBM, set at ~4.8 eV.

Following the second treatment, a high-resolution UPS measurement was made over the 2.8-5.8 eV BE region. Reports of similar scans over the secondary electron peak have provided insight into the details of this peak, namely, energy decline in steps resulting from thermalizing electron-phonon emission [1, 2]. In our case, it instead yielded another interesting result, shown in Figure S5, where the molecular oxygen and lithium-treated sample exhibited a changing electronic structure. This could be a time dependence, although, as it was not observed for the other samples in similar scans, it is more likely caused by the UV photon bombardment during the UPS measurement. This could have an effect similar to molecular oxygen cracking termination methods, where O_2 molecules still present on the surface are dissociated by the UV radiation, changing the chemistry and electronic structure. Only two measurements were conducted on this effect, and we believe further work using different annealing times would provide insight into the process behind the changes occurring.

References:

(1) O'Donnell, K. M.; Edmonds, M. T.; Ristein, J.; Rietwyk, K. J.; Tadich, A.; Thomsen, L.; Pakes, C. I.; Ley, L. Direct observation of phonon emission from hot electrons: spectral features in diamond secondary electron emission. *J. Phys.: Condens. Matter* **2014**, *26* (39), 395008. DOI: 10.1088/0953-8984/26/39/395008.

(2) Wan, G.; Cattelan, M.; Croot, A.; Dominguez-Andrade, H.; Nicley, S. S.; Haenen, K.; Fox, N. A. Spectroscopic insight of low energy electron emission from diamond surfaces. *Carbon* **2021**, *185*, 376-383. DOI: https://doi.org/10.1016/j.carbon.2021.09.045.
